# How We Do Things With Words: Analyzing Text as Social and Cultural Data

**DOI:** 10.3389/frai.2020.00062

**Published:** 2020-08-25

**Authors:** Dong Nguyen, Maria Liakata, Simon DeDeo, Jacob Eisenstein, David Mimno, Rebekah Tromble, Jane Winters

**Affiliations:** ^1^Alan Turing Institute, London, United Kingdom; ^2^Institute for Language, Cognition and Computation, School of Informatics, The University of Edinburgh, Edinburgh, United Kingdom; ^3^Department of Information and Computing Sciences, Utrecht University, Utrecht, Netherlands; ^4^Department of Computer Science, University of Warwick, Coventry, United Kingdom; ^5^School of Electronic Engineering and Computer Science, Queen Mary University of London, London, United Kingdom; ^6^Department of Social and Decision Sciences, Carnegie Mellon University, Pittsburgh, PA, United States; ^7^Santa Fe Institute, Santa Fe, NM, United States; ^8^School of Interactive Computing, Georgia Institute of Technology, Atlanta, GA, United States; ^9^Department of Information Science, Cornell University, Ithaca, NY, United States; ^10^School of Media and Public Affairs, The George Washington University, Washington, DC, United States; ^11^School of Advanced Study, University of London, London, United Kingdom

**Keywords:** computational text analysis, natural language processing, computational social science, cultural analytics, digital humanities

## Abstract

In this article we describe our experiences with computational text analysis involving rich social and cultural concepts. We hope to achieve three primary goals. First, we aim to shed light on thorny issues not always at the forefront of discussions about computational text analysis methods. Second, we hope to provide a set of key questions that can guide work in this area. Our guidance is based on our own experiences and is therefore inherently imperfect. Still, given our diversity of disciplinary backgrounds and research practices, we hope to capture a range of ideas and identify commonalities that resonate for many. This leads to our final goal: to help promote interdisciplinary collaborations. Interdisciplinary insights and partnerships are essential for realizing the full potential of any computational text analysis involving social and cultural concepts, and the more we bridge these divides, the more fruitful we believe our work will be.

## 1. Introduction

In June 2015, the operators of the online discussion site Reddit banned several communities under new anti-harassment rules. Chandrasekharan et al. (2017) used this opportunity to combine rich online data with computational methods to study a current question: Does eliminating these “echo chambers” diminish the amount of hate speech overall? Exciting opportunities like these, at the intersection of “thick” cultural and societal questions on the one hand, and the computational analysis of rich textual data on larger-than-human scales on the other, are becoming increasingly common.

Indeed, computational analysis is opening new possibilities for exploring challenging questions at the heart of some of the most pressing contemporary cultural and social issues. While a human reader is better equipped to make logical inferences, resolve ambiguities, and apply cultural knowledge than a computer, human time and attention are limited. Moreover, many patterns are not obvious in any specific context, but only stand out in the aggregate. For example, in a landmark study, Mosteller and Wallace (1963) analyzed the authorship of *The Federalist Papers* using a statistical text analysis by focusing on style, based on the distribution of function words, rather than content. As another example, Long and So (2016) studied what defines English haiku and showed how computational analysis and close reading can complement each other. Computational approaches are valuable precisely because they help us identify patterns that would not otherwise be discernible.

Yet these approaches are not a panacea. Examining thick social and cultural questions using computational text analysis carries significant challenges. For one, texts are culturally and socially situated. They reflect the ideas, values and beliefs of both their authors and their audiences, and such subtleties of meaning and interpretation are difficult to incorporate in computational approaches. For another, many of the social and cultural concepts we seek to examine are highly contested—hate speech is just one such example. Choices regarding how to operationalize and analyze these concepts can raise serious concerns about conceptual validity and may lead to shallow or obvious conclusions, rather than findings that reflect the depth of the questions we seek to address.

These are just a small sample of the many opportunities and challenges faced in computational analyses of textual data. New possibilities and frustrating obstacles emerge at every stage of research, from identification of the research question to interpretation of the results. In this article, we take the reader through a typical research process that involves measuring social or cultural concepts using computational methods, discussing both the opportunities and complications that often arise. In the Reddit case, for example, hate speech is measured, however imperfectly, by the presence of particular words semi-automatically extracted from a machine learning algorithm. Operationalizations are never perfect translations, and are often refined over the course of an investigation, but they are crucial.

We begin our exploration with the identification of research questions, proceed through conceptualization, data selection, and operationalization, and end with analysis and the interpretation of results. The research process sounds more or less linear this way, but each of these phases overlaps, and in some instances requires us to return to previous steps. The analysis phase, for example, often feeds back into the original research questions, which may continue to evolve for much of the project. At each stage, our discussion is critically informed by insights from the humanities and social sciences, fields that have focused on, and worked to tackle, the challenges of textual analysis—albeit at smaller scales—since their inception.

In describing our experiences with computational text analysis, we hope to achieve three primary goals. First, we aim to shed light on thorny issues not always at the forefront of discussions about computational text analysis methods. Second, we hope to provide a set of key questions that can guide work with thick social and cultural concepts. Our guidance is based on our own experiences and is therefore inherently imperfect. Still, given our diversity of disciplinary backgrounds and research practices, we hope to capture a range of ideas and identify commonalities that will resonate for many. This leads to our final goal: to help promote interdisciplinary collaborations. Interdisciplinary insights and partnerships are essential for realizing the full potential of any computational text analysis that involves social and cultural concepts, and the more we are able to bridge these divides, the more fruitful we believe our work will be.

## 2. Research Questions

We typically start by identifying the questions we wish to explore. Can text analysis provide a new perspective on a “big question” that has been attracting interest for years? Or can we raise new questions that have only recently emerged, for example about social media? For social scientists working in computational analysis, the questions are often grounded in theory, asking: How can we explain what we observe? These questions are also influenced by the availability and accessibility of data sources. For example, the choice to work with data from a particular social media platform may be partly determined by the fact that it is freely available, and this will in turn shape the kinds of questions that can be asked.

Computational analysis of text motivated by these questions is insight driven: we aim to describe a phenomenon or explain how it came about. For example, what can we learn about how and why hate speech is used or how this changes over time? Is hate speech one thing, or does it comprise multiple forms of expression? Is there a clear boundary between hate speech and other types of speech, and what features make it more or less ambiguous? In these cases, it is critical to communicate high-level patterns in terms that are recognizable.

This contrasts with much of the work in computational text analysis, which tends to focus on automating tasks that humans perform inefficiently. These tasks range from the annotation of linguistic features that constitute the backbone of natural language processing (NLP), such as part-of-speech tagging (assigning parts of speech to words), to tasks such as spam filtering and sentiment detection, which are motivated by applications like online content moderation. Success, then, is often measured by performance, and communicating why a certain prediction was made—for example, why a document was labeled as positive sentiment, or why a word was classified as a noun—has traditionally been less important than the accuracy of the prediction itself. While more recent research has focused on building systems whose predictions are “explainable” (Ribeiro et al., 2016) or whose workings are “interpretable” (Doshi-Velez and Kim, 2017; Lipton, 2018), such approaches still center the task of prediction, rather than the generation of insights about relationships between theoretically-motivated constructs from the social sciences and humanities.

Domain experts and fellow researchers can provide feedback on questions and help with dynamically revising them. For example, they may say, “*we already think we know that,” “that's too naïve,” “that doesn't reflect social reality,” “text analysis alone is unlikely to answer that question”* (negative); “*two major camps in the field would give different answers to that question”* (neutral); “*we tried to look at that back in the 1960s, but we didn't have the technology”* (positive); and “*that sounds like something that people who made that archive would love,” “that's a really fundamental question”* (very positive). Domain experts in the social sciences and humanities can also help think through the strengths and weaknesses of using computational methodologies to answer a research question. They may, for example, point to areas where adding qualitative insights would strengthen the computational analysis and lead to a richer answer to the research question.

Sometimes we also hope to explicitly connect our work to multiple disciplines. For example, while focusing on the humanistic concerns of an archive, we could also ask social questions such as “*is this archive more about collaborative processes, culture-building or norm creation?”* or “*how well does this archive reflect the society in which it is embedded?”* Murdock et al. (2017) used quantitative methods to tell a story about Darwin's intellectual development—an essential biographical question for a key figure in the history of science. At the same time, their methods connected Darwin's development to the changing landscape of Victorian scientific culture, allowing them to contrast Darwin's “foraging” in the scientific literature of his time to the ways in which that literature was itself produced. Finally, their methods provided a case study, and validation of technical approaches, for cognitive scientists who are interested in how people explore and exploit sources of knowledge.

Questions about potential “dual use” may also arise. Returning to our introductory example, Chandrasekharan et al. (2017) started with a deceptively simple question: if an internet platform eliminates forums for hate speech, does this impact hate speech in other forums? The research was motivated by the belief that a rising tide of online hate speech was (and is) making the internet increasingly unfriendly for disempowered groups, including minorities, women, and LBGTQ individuals. Yet the possibility of dual use troubled the researchers from the onset. Could the methodology be adopted to target the speech of groups like Black Lives Matter? Could it be adopted by repressive governments to minimize online dissent? While these concerns remained, they concluded that hypothetical dual use scenarios did not outweigh the tangible contribution this research could offer toward making the online environment more equal and just.

## 3. Conceptualization

When considering potential research questions, we also must think carefully about the key social and cultural concepts underlying those questions. For example, previous research has considered concepts such as respect (Voigt et al., 2017), conversational failure (Zhang et al., 2018), folktale types and motifs (Meder et al., 2016), social roles (Yang et al., 2019), literary character (Bamman et al., 2014b), hate speech (Chandrasekharan et al., 2017), and trolling (Cheng et al., 2017). A core step in many analyses involves translating these concepts into measurable quantities. However, before we can develop measurements (the operationalization step, or the “implementation” step as denoted by Piper, 2017), we need to first define the concepts. Yet this is rarely a simple task.

In the conceptualization phase we often start with questions such as: who are the domain experts, and how have they approached the topic? We are looking for a definition of the concept that is flexible enough to apply to the data we expect to use, yet formal enough for computational research. For example, our introductory study on hate speech (Chandrasekharan et al., 2017) used a statement on hate speech produced by the European Union Court of Human Rights. The goal was not to implement this definition directly in software but to use it as a reference point to anchor subsequent analyses.

If we want to move beyond the use of *ad hoc* definitions, it can be useful to distinguish between what political scientists Adcock and Collier (2001) call the “background concept” and the “systematized concept.” The background concept comprises the full and diverse set of meanings that might be associated with a particular term. This involves delving into theoretical, conceptual, and empirical studies to assess how a concept has been defined by other scholars and, most importantly, to determine which definition is most appropriate for the particular research question and the theoretical framework in which it is situated. That definition, in turn, represents the systematized concept: the formulation that is adopted for the study.

It is important to consider that for social and cultural concepts there is no absolute ground truth. There are often multiple valid definitions for a concept (the “background” concept in the terms of Adcock and Collier), and definitions might be contested over time. This may be uncomfortable for natural language processing and machine learning researchers, whose primary measure of success is often based on comparing a model's output against “ground truth” or a “gold standard,” e.g., by comparing a sentiment classifier's output against manual annotations. However, the notion of ground truth is uncommon in the humanities and social sciences and it is often taken too far in machine learning. Kirschenbaum (2007, p. 1) notes that in literary criticism and the digital humanities more broadly “*interpretation, ambiguity, and argumentation are prized far above ground truth and definitive conclusions."* Hammond et al. (2013, p. 2) draw attention to the different attitudes of literary scholars and computational linguists toward ambiguity, stating that “*In Computational Linguistics [.] ambiguity is almost uniformly treated as a problem to be solved; the focus is on disambiguation, with the assumption that one true, correct interpretation exists*." The latter is probably true for tasks such as spam filtering, but in the social sciences and the humanities many relevant concepts are fundamentally unobservable, such as latent traits of political actors (Lowe and Benoit, 2013) or cultural fit in organizations (Srivastava et al., 2018), leading to validation challenges. Moreover, when the ground truth comes from people, it may be influenced by ideological priors, priors, priming, simple differences of opinion or perspective, and many other factors (DiMaggio, 2015). We return to this issue in our discussions on validation and analysis.

## 4. Data

We now decide on the data sources, collect and compile the dataset, and inspect its metadata.

### 4.1. Data Acquisition

Many scholars in the humanities and the social sciences work with sources that are not available in digital form, and indeed may never be digitized. Others work with both analog and digitized materials, and the increasing digitization of archives has opened opportunities to study these archives in new ways. We can go to the canonical archive or open up something that nobody has studied before. For example, we might focus on major historical moments (French Revolution, post-Milosevic Serbia) or critical epochs (Britain entering the Victorian era, the transition from Latin to proto-Romance). Or, we could look for records of how people conducted science, wrote and consumed literature, and worked out their philosophies.

#### 4.1.1. Born-Digital Data

A growing number of researchers work with born-digital sources or data (Salganik, 2017). Born-digital data, e.g., from social media, generally do not involve direct elicitation from participants and therefore enable unobtrusive measurements (Webb et al., 1966; Tangherlini, 2016). In contrast, methods like surveys sometimes elicit altered responses from participants, who might adapt their responses to what they think is expected. Moreover, born-digital data is often massive, enabling large-scale studies of language and behavior in a variety of social contexts.

Still, many scholars in the social sciences and humanities work with multiple data sources. The variety of sources typically used means that more than one data collection method is often required. For example, a project examining coverage of a UK General Election, could draw data from traditional media, web archives, Twitter and Facebook, campaign manifestos, etc. and might combine textual analysis of these materials with surveys, laboratory experiments, or field observations offline. In contrast, many computational studies based on born-digital data have focused on one specific source, such as Twitter.

The use of born-digital data raises ethical concerns. Although early studies often treated privacy as a binary construct, many now acknowledge its complexity (danah boyd and Crawford, 2012). Conversations on private matters can be posted online, visible for all, but social norms regarding what should be considered public information may differ from the data's explicit visibility settings. Often no informed consent has been obtained, raising concerns and challenges regarding publishing content and potentially harmful secondary uses (Salganik, 2017; Williams et al., 2017).

Recently, concerns about potential harms stemming from secondary uses have led a number of digital service providers to restrict access to born-digital data. Facebook and Twitter, for example, have reduced or eliminated public access to their application programming interfaces (APIs) and expressed hesitation about allowing academic researchers to use data from their platforms to examine certain sensitive or controversial topics. Despite the seeming abundance of born-digital data, we therefore cannot take its availability for granted.

#### 4.1.2. Data Quality

Working with data that someone else has acquired presents additional problems related to provenance and contextualization. It may not always be possible to determine the criteria applied during the creation process. For example, why were certain newspapers digitized but not others, and what does this say about the collection? Similar questions arise with the use of born-digital data. For instance, when using the Internet Archive's Wayback Machine to gather data from archived web pages, we need to consider what pages were captured, which are likely missing, and why.

We must often repurpose born-digital data (e.g., Twitter was not designed to measure public opinion), but data biases may lead to spurious results and limit justification for generalization (Olteanu et al., 2019). In particular, data collected via black box APIs designed for commercial, not research, purposes are likely to introduce biases into the inferences we draw, and the closed nature of these APIs means we rarely know what biases are introduced, let alone how severely they might impact our research (Morstatter et al., 2013; Tromble et al., 2017). These, however, are not new problems. Historians, for example, have always understood that their sources were produced within particular contexts and for particular purposes, which are not always apparent to us.

Non-representative data can still be useful for making comparisons within a sample. In the introductory example on hate speech (Chandrasekharan et al., 2017), the Reddit forums do not present a comprehensive or balanced picture of hate speech: the writing is almost exclusively in English, the targets of hate speech are mainly restricted (e.g., to black people, or women), and the population of writers is shaped by Reddit's demographics, which skew toward young white men. These biases limit the generalizability of the findings, which cannot be extrapolated to other languages, other types of hate speech, and other demographic groups. However, because the findings are based on measurements on the same sort of hate speech and the same population of writers, as long as the collected data are representative of this specific population, these biases do not pose an intractable validity problem if claims are properly restricted.

The size of many newly available datasets is one of their most appealing characteristics. Bigger datasets often make statistics more robust. The size needed for a computational text analysis depends on the research goal: When it involves studying rare events, bigger datasets are needed. However, larger is not always better. Some very large archives are “secretly” collections of multiple and distinct processes that no in-field scholar would consider related. For example, Google Books is frequently used to study cultural patterns, but the over-representation of scientific articles in Google books can be problematic (Pechenick et al., 2015). Even very large born-digital datasets usually cover limited timespans compared to, e.g., the Gutenberg archive of British novels.

This stage of the research also raises important questions about fairness. Are marginalized groups, for example, represented in the tweets we have collected? If not, what types of biases might result from analyses relying on those tweets?

Local experts and “informants” can help navigate the data. They can help understand the role an archive plays in the time and place. They might tell us: Is this the central archive, or a peripheral one? What makes it unusual? Or they might tell us how certain underrepresented communities use a social media platform and advise us on strategies for ensuring our data collection includes their perspectives (Frey et al., 2018).

However, when it is practically infeasible to navigate the data in this way—for instance, when we cannot determine what is missing from Twitter's Streaming API or what webpages are left out of the Internet Archive—we should be open about the limitations of our analyses, acknowledging the flaws in our data and drawing cautious and reasonable conclusions from them. In all cases, we should report the choices we have made when creating or re-using any dataset.

### 4.2. Compiling Data

After identifying the data source(s), the next step is compiling the actual data set. The breadth and scope of a dataset define the set of questions that are possible to answer from that dataset. For example, we often have a specific set of documents in mind: an author's work, a particular journal, a time period. But if we want to say that this “core” set has some distinctive property, we need a “comparison” set. Expanding the collection beyond the documents that we would immediately think of has the beneficial effect of increasing our sample size. Having more sources increases the chance that we will notice something consistent across many individually varying contexts. If we do not have sufficient breadth, we cannot support arguments that involve making comparisons.

Comparing sets of documents can sometimes support causal inference, presented as a contrast between a treatment group and a control. In Chandrasekharan et al. (2017), the treatment consisted of the text written in the two forums that were eventually closed by Reddit. However, identifying a control group required a considerable amount of time and effort. Reddit is a diverse platform, with a wide variety of interactional and linguistic styles; it would be pointless to compare hate speech forums against forums dedicated to, say, pictures of wrecked bicycles^1^, and such a comparison would surface many differences that are irrelevant to the original research question. Chandrasekharan et al. used a matching design, populating the control group with forums that were as similar as possible to the treatment group, but were not banned from Reddit. The goal is to estimate the counterfactual scenario: in this case, what would have happened had the site not taken action against these specific forums? An ideal control would make it possible to distinguish the effect of the treatment—closing the forums—from other idiosyncratic properties of texts that were treated.

We also look for categories of documents that might not be useful. We might remove documents that are meta-discourse, like introductions and notes, or documents that are in a language that is not the primary language of the collection, or duplicates when we are working with archived web pages. However, we need to carefully consider the potential consequences of information we remove. Does its removal alter the data, or the interpretation of the data, we are analyzing? Are we losing anything that might be valuable at a later stage?

### 4.3. Labels and Metadata

Sometimes all we have is documents, but often we want to look at documents in the context of some additional information, or metadata. This additional information could tell us about the creation of documents (date, author, forum), or about the reception of documents (flagged as hate speech, helpful review). Information about text segments can be extremely valuable, but it is also prone to errors, inconsistencies, bias, and missing information. Examining metadata is a good way to check a collection's balance and representativeness. Are sources disproportionately of one form? Is the collection missing a specific time window? This type of curation can be extremely time consuming as it may require expert labeling, but it often leads to the most compelling results. Sometimes metadata are also used as target labels to develop machine learning models. But using them as a “ground truth” requires caution. Labels sometimes mean something different than we expect. For example, a down vote for a social media post could indicate that the content is offensive, or that the voter simply disagreed with the expressed view.

## 5. Operationalization

In this phase we develop measures (or, “operationalizations,” or “indicators”) for the concepts of interest, a process called “operationalization.” Regardless of whether we are working with computers, the output produced coincides with Adcock and Collier's “scores”—the concrete translation and output of the systematized concept into numbers or labels (Adcock and Collier, 2001). Choices made during this phase are always tied to the question “Are we measuring what we intend to measure?” Does our operationalization match our conceptual definition? To ensure validity we must recognize gaps between what is important and what is easy to measure. We first discuss modeling considerations. Next, we describe several frequently used computational approaches and their limitations and strengths.

### 5.1. Modeling Considerations

#### Variable types

In many cases, the variables of interest (both predictors and outcomes) are not simply binary or categorical. For example, a study on language use and age could focus on chronological age (instead of, e.g., social age, Eckert, 1997). However, even then, age can be modeled in different ways. Discretization—converting a continuous variable to a discrete variable—can facilitate quantitative analysis when the relationship of interest is non-linear. For example, research in both natural language processing and sociolinguistics has often modeled age as a categorical variable (Eckert, 1997; Nguyen et al., 2016). But any discretization raises questions: How many categories? Where to place the boundaries? Fine distinctions might not always be meaningful for the analysis we are interested in, but categories that are too broad can threaten validity (Royston et al., 2006).

Variables may also have internal structure. For example, spatial location is inherently multidimensional, and must be considered in relation to landmarks and boundaries; social network position is an inherently relational contract; even time, while intrinsically one-dimensional, must often be viewed in relation to an overlapping set of landmarks such as the hours of the conventional workday and the days of the week (Golder and Macy, 2011). Such issues can be handled with discretization, but it is often preferable to keep the variable in its most precise form. For example, while some work on geospatial language variation discretizes to administrative boundaries such as cities (e.g., Grieve et al., 2011) or U.S. census regions (e.g., Eisenstein et al., 2014), such politically-defined units may not correspond to linguistic reality. As an alternative, Nguyen and Eisenstein (2017) work directly with spatial coordinates, using non-parametric hypothesis testing to identify linguistic terms with significant spatial variation. This makes it possible to recognize fine-grained effects, such as language variation across the geography of a city.

#### Categorization scheme

Using a particular classification scheme means deciding which variations are visible, and which ones are hidden (Bowker and Star, 1999). We are looking for a categorization scheme for which it is feasible to collect a large enough labeled document collection (e.g., to train supervised models), but which is also fine-grained enough for our purposes. As Bowker and Star (1999) show, classification schemes rarely exhibit the ideal properties, i.e., that they are consistent, their categories are mutually exclusive, and that the system is complete. Borderline cases are challenging, especially with social and cultural concepts, where the boundaries are often not clear-cut. The choice of scheme can also have ethical implications (D'Ignazio and Klein, 2020). For example, gender is usually represented as a binary variable in NLP; computational models built on this foundation risk learning gender-stereotypical patterns. For this reason, a growing line of research has sought new ways to operationalize gender in NLP (Bamman et al., 2014a; Nguyen et al., 2014; Koolen and van Cranenburgh, 2017).

#### Supervised vs. unsupervised

Supervised and unsupervised learning are the most common approaches to learning from data. With supervised learning, a model learns from *labeled* data (e.g., social media messages labeled by sentiment) to infer (or predict) these labels from unlabeled texts. In contrast, unsupervised learning uses *unlabeled* data. Supervised approaches are especially suitable when we have a clear definition of the concept of interest and when labels are available (either annotated or native to the data). For example, Althoff et al. (2014) build a classifier to predict when an altruistic request is likely to succeed, using annotations that are structurally encoded by a specific social media community; Tan et al. (2016) apply a similar strategy to learn to predict the persuasiveness of textual arguments. While supervised learning can be viewed as a subdomain of machine learning, we note that methods such as regression and classification are part of the standard toolkit of quantitative social science (Hastie et al., 2009), and that such techniques have been applied to text for decades (e.g., Mosteller and Wallace, 1963)^2^.

Unsupervised approaches, such as topic models, uncover natural structure in the data and are therefore especially useful for exploration. For example, Chandrasekharan et al. (2018) identify clusters of content-moderation strategies on Reddit, corresponding to natural groupings of communities based on their moderation stances toward various types of content. In this setting, conceptualization and operationalization may occur simultaneously, with theory emerging from the data (Baumer et al., 2017). Unsupervised approaches are also used when there is a clear way of measuring a concept, often based on strong assumptions. For example, Murdock et al. (2017) measure “surprise” in an analysis of Darwin's reading decisions based on the divergence between two probability distributions.

Unsupervised learning can be combined with supervised learning in more elaborate research designs. In their analysis of the language used by police officers during routine traffic stops, Voigt et al. (2017) first obtained manual annotations for five “conceptually overlapping folk notions related to respect and officer treatment.” They then applied principal component analysis—an unsupervised technique—to identify two independent dimensions of variation among the five original annotations, which they labeled as “respect” and “formality.” Finally, they trained a supervised machine learning system to detect these characteristics at scale, using the initial set of labels as training data.

#### Units of interest

From an analytical perspective, the unit of text that we are labeling (or annotating, or coding), either automatic or manual, can sometimes be different than one's final unit of analysis. Consider the example of sentiment analysis. We often classify a review as positive or negative as a whole, but even the level of individual sentences may be too coarse: “The service was slow and rude, but the potatoes are to die for” requires annotation at the level of clauses or phrases. Another example might be a study on media frames in news stories. If the theoretical framework and research question point toward frames at the story level (e.g., what is the overall causal analysis of the news article?), the story must be the unit of analysis (Entman, 2004). Yet it is often difficult to validly and reliably code a single frame at the story level. Multiple perspectives are likely to sit side-by-side in a story. Thus, an article on income inequality might point to multiple causes, such as globalization, education, and tax policies. Coding at the sentence level would detect each of these causal explanations individually, but this information would need to be somehow aggregated to determine the overall story-level frame. Sometimes scholars solve this problem by examining only headlines (e.g., Aubrey, 2010; Bleich et al., 2015), sometimes arguing that based on journalistic convention and readers' habits, the most important information can be found at the beginning of a story (Bleich et al., 2016). However, this leads to a return to a shorter, less nuanced analysis.

From a computational perspective, the unit of text can also make a huge difference, especially when we are using bag-of-words models, where word order within a unit does not matter (Boyd-Graber et al., 2017). Finding a good segmentation sometimes means combining short documents and subdividing long documents. Small segments, like tweets, sometimes do not have enough information to make their semantic context clear (Mehrotra et al., 2013). In contrast, larger segments, like novels, have too much variation, making it difficult to train focused models (Jockers, 2013). The word “document” can therefore be misleading. But it is so ingrained in the common NLP lexicon that we use it anyway in this article.

#### Interpretability

For insight-driven text analysis, it is often critical that high-level patterns can be communicated. Furthermore, interpretable models make it easier to find spurious features, to do error analysis, and to support interpretation of results. Some approaches are effective for prediction, but harder to interpret. The value we place on interpretability can therefore influence the approach we choose. There is an increasing interest in developing interpretable or transparent models in the NLP and machine learning communities, as evidenced by new venues such as the ACM Conference on Fairness, Accountability, and Transparency. However, the concept of interpretability is difficult to place on a firm theoretical footing (Lipton, 2018), and may only be tractable when viewed from a multidimensional perspective (Doshi-Velez and Kim, 2017).

### 5.2. Annotation

Many studies involve human coders. Sometimes the goal is to fully code the data, but in a computational analysis we often use the labels (or annotations) to train machine learning models to automatically recognize them, and to identify language patterns that are associated with these labels. For example, for a project analyzing rumors online (Zubiaga et al., 2016b), conversation threads were annotated along different dimensions, including rumor vs. non-rumor and stance toward a rumor.

The collection of annotation choices make up an annotation scheme (or “codebook”). Existing schemes and annotations can be useful as starting points. Usually settling on an annotation scheme requires several iterations, in which the guidelines are updated and annotation examples are added. For example, a political scientist could use a mixed deductive-inductive strategy for developing a codebook. She starts by laying out a set of theory-driven deductive coding rules, which means that the broad principles of the coding rules are laid out without examining examples first. These are then tested (and possibly adjusted) based on a sample of the data. In line with Adcock and Collier's notion of “content validity” (Adcock and Collier, 2001), the goal is to assess whether the codebook adequately captures the systematized concept. By looking at the data themselves, she gains a better sense of whether some things have been left out of the coding rules and whether anything is superfluous, misleading, or confusing. Adjustments are made and the process is repeated, often with another researcher involved.

The final annotations can be collected using a crowdsourcing platform, a smaller number of highly-trained annotators, or a group of experts. Which type of annotator to use should be informed by the complexity and specificity of the concept. For more complex concepts, highly-trained or expert annotators tend to produce more reliable results. However, complex concepts can sometimes be broken down into simpler micro-tasks, and annotations can sometimes be made more reliable by aggregating across multiple crowd workers (Snow et al., 2008). Concepts from highly specialized domains, such as theoretical syntax, may also require expert annotators. In all cases, however, some training will be required, and the training phase should involve continual checks of inter-annotator agreement (i.e., intercoder reliability) or checks against a gold standard (e.g., quizzes in crowdsourcing platforms).

Researchers must also decide how inter-annotator agreement will be measured and what an acceptable level of agreement would be. Krippendorff's alpha is frequently used in the social sciences, but the right measure depends on the type of data and task. For manual coding, we can continually check inter-annotator agreement and begin introducing checks of *intra*-annotator agreement, too. For most communication scholars using only manual content analysis, by convention an acceptable rate of agreement is achieved when Krippendorf's alpha reaches 0.80 or above (Neuendorf, 2017). When human-coded data are used to validate machine learning algorithms, the reliability of the human-coded data is even more important. Disagreement between annotators can signal weaknesses of the annotation scheme, or highlight the inherent ambiguity in what we are trying to measure. Disagreement itself can be meaningful and can be integrated in subsequent analyses (Aroyo and Welty, 2013; Demeester et al., 2016).

This stage of research also involves considering whether biases could have been introduced in the annotation process. For example, Sap et al. (2019) found racial bias in automatic hate speech detection models. African American English (AAE) tweets and tweets by self-identified African Americans were more likely to be labeled as offensive. However, they showed that when annotators were asked to consider the dialect and race of Twitter users, they were less likely to annotate AAE tweets as offensive.

### 5.3. Data Preprocessing

Preparing the data can be a complex and time-consuming process, often involving working with partially or wholly unstructured data. The pre-processing steps have a big impact on the operationalizations, subsequent analyses and reproducibility efforts (Fokkens et al., 2013), and they are usually tightly linked to what we intend to measure. Unfortunately, these steps tend to be underreported, but documenting the pre-processing choices made is essential and is analogous to recording the decisions taken during the production of a scholarly edition or protocols in biomedical research. Data may also vary enormously in quality, depending on how it has been generated. Many historians, for example, work with text produced from an analog original using Optical Character Recognition (OCR). Often, there will be limited information available regarding the accuracy of the OCR, and the degree of accuracy may even vary within a single corpus (e.g., where digitized text has been produced over a period of years, and the software has gradually improved). The first step, then, is to try to correct for common OCR errors. These will vary depending on the type of text, the date at which the “original” was produced, and the nature of the font and typesetting.

One step that almost everyone takes is to tokenize the original character sequence into the words and word-like units. Tokenization is a more subtle and more powerful process than people expect. It is often done using regular expressions or scripts that have been circulating within the NLP community. Tokenization heuristics, however, can be badly confused by emoticons, creative orthography (e.g., U$A, sh!t), and missing whitespace. Multi-word terms are also challenging. Treating them as a single unit can dramatically alter the patterns in text. Many words that are individually ambiguous have clear, unmistakable meanings as terms, like “black hole” or “European Union.” However, deciding what constitutes a multi-word term is a difficult problem. In writing systems like Chinese, tokenization is a research problem in its own right.

Beyond tokenization, common steps include lowercasing, removing punctuation, stemming (removing suffixes, e.g., mapping “complete” to “complet”), lemmatization (converting inflections to a base lemma, e.g., mapping both “sang” and “sung” to “sing”), and normalization, which has never been formally defined^3^, but often includes grouping abbreviations like “U.S.A.” and “USA,” ordinals like “1st” and “first,” and variant spellings like “noooooo” (Han and Baldwin, 2011). The main goal of these steps is to improve the ratio of tokens (individual occurrences) to types (the distinct things in a corpus). Each step requires making additional assumptions about which distinctions are relevant: is “apple” different from “Apple”? Is “burnt” different from “burned”? Is “cool” different from “coooool”? Sometimes these steps can actively hide useful patterns, like social meaning (Eisenstein, 2013). Some of us therefore try do as little modification as possible.

From a multilingual perspective, English and Chinese have unusually simple inflectional systems, and so it is statistically reasonable to treat each inflection as a unique word type. Romance languages have considerably more inflections than English; many indigenous North American languages have still more. For these languages, unseen data is far more likely to include previously-unseen inflections, and therefore, dealing with inflections is more important. On the other hand, the resources for handling inflections vary greatly by language, with European languages dominating the attention of the computational linguistics community thus far. Current state-of-the-art techniques in NLP address these issues by applying statistical segmentation techniques to whitespace-delimited tokens, yielding a sequence of “word pieces” to be used for all downstream processing (Kudo and Richardson, 2018; Devlin et al., 2019). Word pieces do not necessarily correspond to linguistically meaningful units such as inflectional affixes; furthermore, languages like Arabic employ systems of morphology that cannot be captured by segmentation (Soudi et al., 2007). Thus, while this style of segmentation is sufficient for highly accurate prediction in many tasks, it may not be suitable in cases where interpretability of specific linguistic units is essential.

We sometimes also remove words that are not relevant to our goals, for example by calculating vocabulary frequencies. We construct a “stoplist” of words that we are not interested in. If we are looking for semantic themes we might remove function words like determiners and prepositions. If we are looking for author-specific styles, we might remove all words except function words. Some words are generally meaningful but too frequent to be useful within a specific collection. The word “prisoner” would be very interesting in most contexts, but in London court records that consist entirely of decisions about prisoners, it adds nothing. We sometimes also remove very infrequent words. Their occurrences are too low for robust patterns and removing them helps reducing the vocabulary size.

The choice of processing steps can be guided by theory or knowledge about the domain as well as experimental investigation. When we have labels, predictive accuracy of a model is a way to assess the effect of the processing steps. In unsupervised settings, it is more challenging to understand the effects of different steps. Inferences drawn from unsupervised settings can be sensitive to pre-processing choices (Denny and Spirling, 2018). Stemming has been found to provide little measurable benefits for topic modeling and can sometimes even be harmful (Schofield and Mimno, 2016). All in all, this again highlights the need to document these steps.

Finally, we can also mark up the data, e.g., by identifying entities (people, places, organizations, etc.) or parts of speech (noun, verb, etc.). Although many NLP tools are available for such tasks, they are often challenged by linguistic variation, such as orthographic variation in historical texts (Piotrowski, 2012) and social media (Eisenstein, 2013). Moreover, the performance of NLP tools often drops when applying them outside the training domain, such as applying tools developed on newswire texts to texts written by younger authors (Hovy and Søgaard, 2015). Problems (e.g., disambiguation in named entity recognition) are sometimes resolved using considerable manual intervention. This combination of the automated and the manual, however, becomes more difficult as the scale of the data increases, and the “certainty” brought by the latter may have to be abandoned.

### 5.4. Dictionaries

Dictionaries are frequently used to code texts in content analyses (Neuendorf, 2017). Dictionaries consist of one or more categories (i.e., word lists). Sometimes the output is simply the number of category occurrences (e.g., positive sentiment), thus weighting words within a category equally. In some other cases, words are assigned continuous scores. The high transparency of dictionaries makes them sometimes more suitable than supervised machine learning models. However, dictionaries should only be used if the scores assigned to words match how the words are used in the data (see Grimmer and Stewart, 2013 for a detailed discussion on limitations). There are many off-the-shelf dictionaries available (e.g., LIWC, Tausczik and Pennebaker, 2010). These are often well-validated, but applying them on a new domain may not be appropriate without additional validation. Corpus- or domain-specific dictionaries can overcome limitations of general-purpose dictionaries.

The dictionaries are often manually compiled, but increasingly they are constructed semi-automatically (e.g., Fast et al., 2016). When we semi-automatically create a word list, we use automation to identify an initial word list, and human insight to filter it. By automatically generating the initial words lists, words can be identified that human annotators might have difficulty intuiting. By manually filtering the lists, we use our theoretical understanding of the target concept to remove spurious features.

In the introduction study, SAGE (Eisenstein et al., 2011) was used to obtain a list of words that distinguished the text in the treatment group (subreddits that were closed by Reddit) from text in the control group (similar subreddits that were not closed). The researchers then returned to the hate speech definition provided by the European Court of Human Rights, and manually filtered the top SAGE words based on this definition. Not all identified words fitted the definition. The others included: the names of the subreddits themselves, names of related subreddits, community-specific jargon that was not directly related to hate speech, and terms such as *IQ* and *welfare*, which were frequently used in discourses of hate speech, but had significant other uses. The word lists provided the measurement instrument for their main result, which is that the use of hate speech throughout Reddit declined after the two treatment subreddits were closed.

### 5.5. Supervised Models

Supervised learning is frequently used to scale up analyses. For example, Nguyen et al. (2015) wanted to analyze the motivations of Movember campaign participants. By developing a classifier based on a small set of annotations, they were able to expand the analysis to over 90k participants.

The choice of supervised learning model is often guided by the task definition and the label types. For example, to identify stance toward rumors based on sequential annotations, an algorithm for learning from sequential (Zubiaga et al., 2016a) or time series data (Lukasik et al., 2016) could be used. The features (sometimes called variables or predictors) are used by the model to make the predictions. They may vary from content-based features such as single words, sequences of words, or information about their syntactic structure, to meta-information such as user or network information. Deciding on the features requires experimentation and expert insight and is often called feature engineering. For insight-driven analysis, we are often interested in *why* a prediction has been made and features that can be interpreted by humans may be preferred. Recent neural network approaches often use simple features as input (such as word embeddings or character sequences), which requires less feature engineering but make interpretation more challenging.

Supervised models are powerful, but they can latch on to spurious features of the dataset. This is particularly true for datasets that are not well-balanced, and for annotations that are noisy. In our introductory example on hate speech in Reddit (Chandrasekharan et al., 2017), the annotations are automatically derived from the forum in which each post appears, and indeed, many of the posts in the forums (subreddits) that were banned by Reddit would be perceived by many as hate speech. But even in banned subreddits, not all of the content is hate speech (e.g., some of the top features were self-referential like the name of the subreddit) but a classifier would learn a high weight for these features.

Even when expert annotations are available on the level of individual posts, spurious features may remain. Waseem and Hovy (2016) produced expert annotations of hate speech on Twitter. They found that one of the strongest features for sexism is the name of an Australian TV show, because people like to post sexist comments about the contestants. If we are trying to make claims about what inhibits or encourages hate speech, we would not want those claims to be tied to the TV show's popularity. Such problems are inevitable when datasets are not well-balanced over time, across genres, topics, etc. Especially with social media data, we lack a clear and objective definition of “balance” at this time.

Recent work on explaining decisions of machine learning models can help identify spurious features (Ribeiro et al., 2016; Lapuschkin et al., 2019). Furthermore, placing more emphasis on explainability and interpretability could increase the adoption of supervised learning models for insight-driven analyses. One way would be to only use models that are already somewhat interpretable, for example models that use a small number of human-interpretable features. Rather than imposing such restrictions, there is also work on generating *post-hoc* explanations for individual predictions (e.g., Ribeiro et al. (2016)), even when the underlying model itself is very complex. However, a concern with *post-hoc* explanations is that they may not faithfully reflect the behavior of the original model (Rudin, 2019).

### 5.6. Topic Modeling

Topic models (e.g., LDA, Blei et al., 2003) are usually unsupervised and therefore less biased toward human-defined categories. They are especially suited for insight-driven analysis, because they are constrained in ways that make their output interpretable. Although there is no guarantee that a “topic” will correspond to a recognizable theme or event or discourse, they often do so in ways that other methods do not. Their easy applicability without supervision and ready interpretability make topic models good for exploration. Topic models are less successful for many performance-driven applications. Raw word features are almost always better than topics for search and document classification. LSTMs and other neural network models are better as language models. Continuous word embeddings have more expressive power to represent fine-grained semantic similarities between words.

A topic model provides a different perspective on a collection. It creates a set of probability distributions over the vocabulary of the collection, which, when combined together in different proportions, best match the content of the collection. We can sort the words in each of these distributions in descending order by probability, take some arbitrary number of most-probable words, and get a sense of what (if anything) the topic is “about.” Each of the text segments also has its own distribution over the topics, and we can sort these segments by their probability within a given topic to get a sense of how that topic is used.

One of the most common questions about topic models is how many topics to use, usually with the implicit assumption that there is a “right” number that is inherent in the collection. We prefer to think of this parameter as more like the scale of a map or the magnification of a microscope. The “right” number is determined by the needs of the user, not by the collection. If the analyst is looking for a broad overview, a relatively small number of topics may be best. If the analyst is looking for fine-grained phenomena, a larger number is better.

After fitting the model, it may be necessary to circle back to an earlier phase. Topic models find consistent patterns. When authors repeatedly use a particular theme or discourse, that repetition creates a consistent pattern. But other factors can also create similar patterns, which look as good to the algorithm. We might notice a topic that has highest probability on French stopwords, indicating that we need to do a better job of filtering by language. We might notice a topic of word fragments, such as “*ing*,” “*tion*,” “*inter*,” indicating that we are not handling end-of-line hyphenation correctly. We may need to add to our stoplist or change how we curate multi-word terms.

### 5.7. Validation

The output of our measurement procedures (in the social sciences often called the “scores”) must now be assessed in terms of their reliability and validity with regard to the (systemized) concept. Reliability aims to capture repeatability, i.e., the extent to which a given tool provides consistent results.

Validity assesses the extent to which a given measurement tool measures what it is supposed to measure. In NLP and machine learning, most models are primarily evaluated by comparing the machine-generated labels against an annotated sample. This approach presumes that the human output is the “gold standard” against which performance should be tested. In contrast, when the reliability is measured based on the output of different annotators, no coder is taken as the standard and the likelihood of coders reaching agreement by chance (rather than because they are “correct”) is factored into the resulting statistic. Comparing against a “gold standard” suggests that the threshold for human inter- and intra-coder reliability should be particularly high.

Accuracy, as well as other measures such as precision, recall and F-score, are sometimes presented as a measure of validity, but if we do not have a genuinely objective determination of what something is supposed measure—as is often the case in text analysis—then accuracy is perhaps a better indication of reliability than of validity. In that case, validity needs to be assessed based on other techniques like those we discuss later in this section. It is also worth asking what level of accuracy is sufficient for our analysis and to what extent there may be an upper bound, especially when the labels are native to the data or when the notion of a “gold standard” is not appropriate.

For some in the humanities, validation takes the form of close reading, not designed to confirm whether the model output is correct, but to present what Piper (2015, p. 67–68) refers to as a form of “*further discovery in two directions*.” Model outputs tell us something about the texts, while a close reading of the texts alongside those outputs tells us something about the models that can be used for more effective model building. Applying this circular, iterative process to 450 18th-century novels written in three languages, Piper was able to uncover a new form of “*conversional novel*” that was not previously captured in “*literary history's received critical categories*” (Piper, 2015, p. 92).

Along similar lines, we can subject both the machine-generated output and the human annotations to another round of content validation. That is, take a stratified random sample, selecting observations from the full range of scores, and ask: Do these make sense in light of the systematized concept? If not, what seems to be missing? Or is something extraneous being captured? This is primarily a qualitative process that requires returning to theory and interrogating the systematized concept, indicators, and scores together. This type of validation is rarely done in NLP, but it is especially important when it is difficult to assess what drives a given machine learning model. If there is a mismatch between the scores and systematized concept at this stage, the codebook may need to be adjusted, human coders retrained, more training data prepared, algorithms adjusted, or in some instances, even a new analytical method adopted.

Other types of validation are also possible. For example, we can compare our output with other approaches that aim to capture the same concept or with external measures, such as public opinion polls (O'Connor et al., 2010). In some cases, experiments on synthetic data can allow for controlled comparisons (Nguyen and Eisenstein, 2017; Shoemark et al., 2019). We can also go beyond only evaluating the labels (or point estimates). Lowe and Benoit (2013) used human judgments to not only assess the positional estimates from a scaling method of latent political traits but also to assess uncertainty intervals. Using different types of validation can increase our confidence in the approach, especially when there is no clear notion of ground truth.

Besides focusing on rather abstract evaluation measures, we could also assess the models in task-based settings using human experts. Furthermore, for insight-driven analyses, it can be more useful to focus on improving explanatory power than making small improvements in predictive performance.

## 6. Analysis

In this phase, we use our models to explore or answer our research questions. For example, given a topic model we can look at the connection between topics and metadata elements. Tags such as “hate speech” or metadata information imply a certain way of organizing the collection. Computational models provide another organization, which may differ in ways that provide more insight into how these categories manifest themselves, or fail to do so.

Moreover, when using a supervised approach, the “errors,” i.e., disagreement between the system output and human-provided labels, can point toward interesting cases for closer analysis and help us reflect on our conceptualizations. In the words of Long and So (2016), they can be “*opportunities for interpretation*.” Other types of “failures” can be insightful as well. Sometimes there is a “*dog that didn't bark*” (Doyle, 1892)—i.e., something that everyone thinks we should have found, but we did not. Or, sometimes the failures are telling us about the existence of something in the data that nobody noticed, or thought important, until then (e.g., the large number of travel journals in Darwin's reading lists).

Computational text analysis is not a replacement for but rather an addition to the approaches one can take to analyze social and cultural phenomena using textual data. By moving back and forth between large-scale computational analyses and small-scale qualitative analyses, we can combine their strengths so that we can identify large-scale and long-term trends, but also tell individual stories. For example, the Reddit study on hate speech (Chandrasekharan et al., 2017) raised various follow-up questions: Can we distinguish hate speech from people talking about hate speech? Did people find new ways to express hate speech? If so, did the total amount of online hate speech decrease after all? As possible next steps, a qualitative discourse analyst might examine a smaller corpus to investigate whether commenters were indeed expressing hate speech in new ways; a specialist in interview methodologies might reach out to commenters to better understand the role of online hate speech in their lives. Computational text analysis represents a step toward better understanding social and cultural phenomena, and it is in many cases better suited toward opening questions rather than closing them.

## 7. Conclusion

Insight-driven computational analysis of text is becoming increasingly common. It not only helps us see more broadly, it helps us see subtle patterns more clearly and allows us to explore radical new questions about culture and society. In this article we have consolidated our experiences, as scholars from very different disciplines, in analyzing text as social and cultural data and described how the research process often unfolds. Each of the steps in the process is time-consuming and labor-intensive. Each presents challenges. And especially when working across disciplines, the research often involves a fair amount of discussion—even negotiation—about what means of operationalization and approaches to analysis are appropriate and feasible.

Below, we provide a set of questions, though unavoidably incomplete, that can serve as a guide for thinking through challenges in each step of the research process. These questions complement recent work providing guidance and suggestions about datasets (Bender and Friedman, 2018; Gebru et al., 2018) and models (Mitchell et al., 2019) more specifically. The issues our questions point to are complex, and as new research projects unfold, the answers to these questions may not be readily apparent or simple. Yet we hope that with some thoughtfulness and perseverance, conceptually sound and meaningful work will result.

## Guiding Questions

### Research questions: Is this an interesting problem to study?

- Who is waiting for the answer to your question? What would knowing the answer change, both in your field of study and the wider world?
- Are these questions answerable with text? Are they answerable only or primarily with text? Conversely, are you missing something when you focus on text alone?
- Why is computational text analysis necessary or valuable for answering the research questions?
- If the research question focuses on model performance, what is the added benefit of testing the model on social or cultural textual data?
- To what other disciplines does this research connect? To whom should you turn for further insights on the research questions you're asking?
- Do you have access to data that will support these research questions?
- Have you considered the ethical implications of your research? Who will be affected by decisions made based on your results?

### Conceptualization: What is this all about?

- What are the core concepts you are addressing? And are you being true to their core meaning?
- What are competing definitions? Which is best suited to the task and why?
- Does the systematized concept you've selected reflect an adequate understanding of the background concept?
- How do domain experts approach the topic? Does your research connect to this wider context? Have you considered relevant methods and theories in other domains?
- Is it possible to speak of “ground truth” for the concept(s) in question?

### Data: Is the data suitable to answer the question asked?

- Are sources representative? Are they disproportionately of one form? Are all relevant time windows covered? Does the data represent all relevant groups, including those often marginalized?
- When metadata is available: Are there errors, inconsistencies, biases, or missing information? Is this quality of metadata consistent across the dataset, or are some parts better or worse?
- When labels are available: How were the labels created? Do the labels actually mean what you are using them to represent?
- If you are filtering, subsampling, or selecting from the original data, is the remaining subset representative? Can you describe how selective removal alters the data and the interpretation of the data? Are you losing anything that might be valuable at a later stage?
- Who created the data, and do they have agency over its use? Should this data be used for research? How does respect for document creators affect how you conduct and share your research?

### Operationalization: How do you measure your core concept(s)?

- Which units of text are most suited to capturing the concepts?
- Which textual pre-processing steps are appropriate for your task and data? What information gets lost with each pre-processing step, and what is gained? What errors may be introduced?
- What types of variables best capture the concept? Do they have inherent structure?
- Can unsupervised methods like clustering and topic models reveal relevant structure?
- Does your annotation scheme or codebook adequately capture the systematized concept? How do you place conceptual boundaries, and how do you handle borderline cases? Who is best suited to provide the annotations?
- How to get from text strings to features that are suitable for computation? Do you prefer features that are interpretable by humans? Do you prefer features that are linguistically meaningful? Are there existing dictionaries (lexicons) that can capture the concepts at word/phrase level?
- When you are using existing text processing tools or methods: What data were they developed on? Can you expect them to work well on your data?
- Does your method measure what it is supposed to measure? What types of validation are needed?
- Is something extraneous being captured? Does the model latch on to spurious signals, like words or other signals that correlate with your labels? Are errors distributed evenly, or do the computational methods work better for some types of texts or writers?

### Analysis: What is/are the data telling you?

- Where does your text analysis agree and disagree with human intuitions? Do disagreements tell you about weaknesses of the algorithms, do they highlight interesting edge cases that defy operationalization, or do they reveal that the proposed operationalization was flawed to begin with?
- If it is not possible to make sources representative or when the errors are not distributed evenly, how should this bias be factored into conclusions from the resulting analyses?
- What new questions does your analysis raise? Can engaging with researchers from other disciplines or domain experts help with the interpretation of your findings?

## Author Contributions

DN and ML provided the initial idea for an article aiming to integrate perspectives from different disciplines on analyzing text as social and cultural data. All authors contributed texts written from their perspective. DN then integrated these contributions into a manuscript. All authors further edited and helped shape the final manuscript.

## Conflict of Interest

The authors declare that the research was conducted in the absence of any commercial or financial relationships that could be construed as a potential conflict of interest.

## Footnotes

^1^https://www.reddit.com/r/bustedcarbon/

^2^For a discussion of the differences between the machine learning and statistical viewpoints of these techniques, see Breiman (2001).

^3^Sproat et al. (2001) make a good first step, but this work focuses mainly on speech transcripts rather than social media writing. As a result, it does not include many things that are considered normalization today.
